# Current understanding of allergic transfusion reactions: incidence, pathogenesis, laboratory tests, prevention and treatment

**DOI:** 10.1111/bjh.12150

**Published:** 2012-12-06

**Authors:** Fumiya Hirayama

**Affiliations:** Japanese Red Cross Kinki Block Blood CentreIbaraki-City, Osaka, Japan

**Keywords:** allergic transfusion reaction, IgE, tryptase, basophil activation test, washed platelets

## Abstract

Non-haemolytic transfusion reactions are the most common type of transfusion reaction and include transfusion-related acute lung injury, transfusion-associated circulatory overload, allergic reactions, febrile reactions, post-transfusion purpura and graft-versus- host disease. Although life-threatening anaphylaxis occurs rarely, allergic reactions occur most frequently. If possible, even mild transfusion reactions should be avoided because they add to patients' existing suffering. During the last decade, several new discoveries have been made in the field of allergic diseases and transfusion medicine. First, mast cells are not the only cells that are key players in allergic diseases, particularly in the murine immune system. Second, it has been suggested that immunologically active undigested or digested food allergens in a donor's blood may be transferred to a recipient who is allergic to these antigens, causing anaphylaxis. Third, washed platelets have been shown to be effective for preventing allergic transfusion reactions, although substantial numbers of platelets are lost during washing procedures, and platelet recovery after transfusion may not be equivalent to that with unwashed platelets. This review describes allergic transfusion reactions, including the above-mentioned points, and focusses on their incidence, pathogenesis, laboratory tests, prevention and treatment.

## Incidence

Although reports revealed that allergic reactions with platelets (PLTs) and red blood cells (RBCs) have an incidence rate of 3·7% and 0·15%, respectively, a review of the literature showed that the incidence rate varied by more than 100-fold, probably because of differences in pre-medication use, patient characteristics, product manufacturing, storage time, reporting rates, reaction definitions and monitoring standards (Geiger & Howard, 2007). A Canadian group showed similar allergic reaction incidences with PLTs and RBCs and an allergic reaction incidence of 0·19% with plasma transfusions (Kleinman *et al*, 2003). Thus, transfusions with PLTs are apparently associated with a higher risk than those with other components, although it is unknown whether these differences are due to the nature of each component or patient factors, including background diseases and history of previous transfusions.

## Allergen-dependent pathways

### Plasma proteins as allergens

The allergens that cause allergic transfusion reactions are plasma proteins such as IgA (Vyas *et al*, 1968; Schmidt *et al*, 1969; Sandler *et al*, 1995) and haptoglobin (Hp) (Koda *et al*, 2000; Shimada *et al*, 2002). Although there are case reports of anaphylactic reactions, many of which were serious (Vyas *et al*, 1968; Schmidt *et al*, 1969; Sandler *et al*, 1995; Koda *et al*, 2000; Shimada *et al*, 2002), there are no reliable estimates regarding the incidence of IgA- and Hp-mediated allergic transfusion reactions. The number of patients who have IgA- or Hp-deficiency and specific antibodies but are never diagnosed because of the absence of symptoms after transfusion remains unknown. Therefore, at least with regard to IgA deficiency, reactions usually do not occur and anaphylaxis appears to be relatively rare (Sandler, 2006).

Although most IgA-related anaphylactic reactions occur in those who are IgA-deficient (serum IgA <0·5 mg/l) and in whom there are detectable serum class-specific IgA antibodies, there are patients with normal serum concentrations of IgA and subclass (IgA1 or IgA2)- or allotype [IgA2m(1) or IgA2m(2)]-specific IgA antibodies who have experienced severe acute reactions to blood transfusions (Sandler *et al*, 1995).

Plasma Hp levels should be carefully measured because they are known to decrease to below detectable levels in certain pathological conditions, such as haemolysis and liver dysfunction (Rougemont *et al*, 1980). In these instances, a DNA diagnosis of Hp deficiency is useful (Koda *et al*, 2000).

Of note, evident racial differences in the incidence of IgA- and Hp-deficiency and consequent racial differences in the prevalence of anaphylactic shock mediated by these antibodies have been observed. The *HP*^*del*^ allele frequency in East and South-east Asian populations is 1·5–3%. Therefore, the incidence of its deficiency is 1/1 000 to 1/4 000. However, this allele has not been detected in African, Western and Southern Asian or European populations (Koda *et al*, 2000; Shimada *et al*, 2007; Soejima *et al*, 2007). In contrast, the incidence of IgA deficiency among the Japanese was reported to be approximately 1/30 000, which was lower than that reported among Europeans (1/2 500) (Ropars *et al*, 1982; Kanoh *et al*, 1986). Therefore, Hp-deficiency and Hp antibodies should be considered among Eastern Asians, while IgA deficiency and IgA antibodies should be considered among Europeans, respectively, as the cause of transfusion-related anaphylactic reactions.

Although rare, anaphylactic shock after transfusions has been reported in complement C4-deficient (Lambin *et al*, 1984; Westhoff *et al*, 1992) and von Willebrand factor-deficient (Bergamaschini *et al*, 1995) patients. Factor IX inhibitors in haemophilia B patients occasionally induce anaphylactic shock after Factor IX transfusions (Warrier & Lusher, 1998).

### Chemical allergens

Besides plasma proteins, methylene blue, which is a reagent used for viral inactivation of fresh frozen plasma, has been reported to cause anaphylactic shock after plasma transfusion (Dewachter *et al*, 2011; Nubret *et al*, 2011). Although the risk appears to be extremely low in light of the widespread use of this blood component, precautions need to be taken in countries and regions that have a methylene blue inactivation system.

### Food allergens

Recently, an intriguing case report suggested that transfusion-related anaphylaxis occurred after passive transfer of peanut allergens to a sensitized person (Jacobs *et al*, 2011). Briefly, a 6-year-old boy had an anaphylactic reaction with rash, angioedoema, hypotension and difficult breathing during a PLT transfusion. Laboratory tests ruled out possible deficiencies in IgA, Hp and C4; allergies to drugs or latex; the presence of human leucocyte antigen (HLA) antibodies and transfusion-associated acute lung injury (TRALI). However, the patient had a history of serious peanut allergy at the age of 1 year and his serum contained specific IgE antibodies against the major peanut allergen Ara h2, which is very resistant to digestion by pepsin. In addition, three of five blood donors recalled having eaten several handfuls of peanuts the evening before they donated blood. This circumstantial evidence suggested that an anaphylactic reaction occurred after the passive transfer of peanut allergens. This is consistent with the fact that substantial amounts of food antigens are absorbed into the blood circulation in a digested form but retain a certain molecular size and antigenicity (Untersmayr & Jensen-Jarolim, 2008). The direct way to prove this hypothesis would be to show these allergens in the transfused blood. However, no data demonstrating the presence of peanut allergens in the donors' blood were provided. The authors only referred to a previous report, which showed that a digestion-resistant peptide of Ara h2 was detected in the serum for up to 24 h after ingestion (Baumert *et al*, 2010). In addition, other investigators have unsuccessfully attempted to detect any circulating peanut proteins in volunteers after very large ingestions (Vickery *et al*, 2011). There is scepticism regarding the assumption that physiologically relevant contamination had occurred when only three of five donors had consumed several handfuls of peanuts the evening before they donated blood (Vickery *et al*, 2011). The true incidence of these reactions is obscure. Therefore, we need to assume a cautious attitude towards this issue until more concrete evidence is provided.

### IgE-, FcεR-, mast cell- and histamine-mediated sub-pathway versus IgG-, FcγRs-, basophil- and platelet-activating factor (PAF)-mediated sub-pathway

**Fig. 1:**
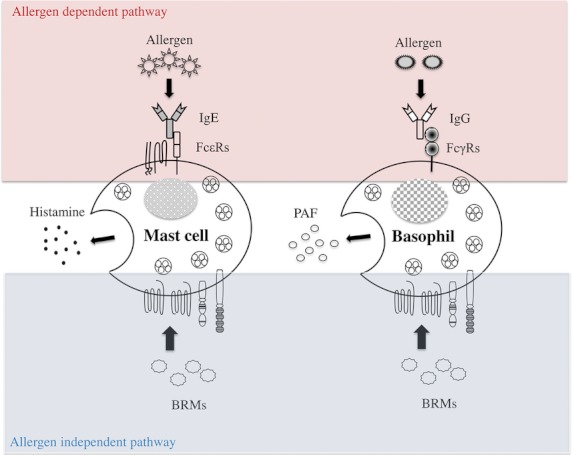
Allergen-dependent and -independent pathways and mast cell-mediated and basophil-mediated sub-pathways in the murine immune system. The allergen-dependent pathway is triggered when allergens bind to antibodies that are bound by FcRs expressed on mast cells and basophils. Subsequently, activated mast cells and basophils release chemical mediators. The allergen-independent pathway is triggered when biological response modifiers (BRMs) bind to their respective receptors expressed on mast cells and basophils, which results in the activation of these cells. In the allergen-dependent mast cell-mediated sub-pathway, IgE and FcεR come into play and histamine is released. In contrast, in the allergen-dependent basophil-mediated sub-pathway, IgG and FcγRs come into play and platelet-activating factor (PAF) is released. Neutrophils and monocytes are reported to be other key players in allergy. IgG and FcγRs are involved and PAF is released.

IgG antibodies are usually detected in both IgA- and Hp-mediated anaphylaxis, while IgE antibodies have also been detected in several studies (Burks *et al*, 1986; Harper *et al*, 1995; Dioun *et al*, 1998; Shimada *et al*, 2002). Therefore, both IgG- and IgE-mediated mechanisms are possible. In cases of anaphylaxis, it is important to examine for IgG antibodies and, if possible, the IgE class. Although IgE, FcεR, mast cells and histamine are considered to play major roles in anaphylaxis, IgG-mediated systemic anaphylaxis was recently demonstrated in the murine system involving FcγRs, basophils and PAF as major players (Fig 1). In this reaction, PAF rather than histamine was the major chemical mediator that induced systemic anaphylaxis (Tsujimura *et al*, 2008). Although it remains uncertain whether this pathway is present in humans, there is supportive evidence for this mechanism.

For example, allergen-specific IgG antibodies rather than IgE antibodies were detected in individuals who manifested systemic anaphylaxis against medical reagents, such as protamine, dextran and recombinant IgG, including anti-tumour necrosis factor-α (Kraft *et al*, 1982; Weiss *et al*, 1989; Adourian *et al*, 1993; Cheifetz *et al*, 2003). Vadas *et al* (2008) investigated the roles of PAF and PAF acetylhydrolase, the enzyme that inactivates PAF, in anaphylaxis in humans. They concluded that a failure of PAF acetylhydrolase to inactivate PAF may contribute to the severity of anaphylaxis on the basis of the following observations: (i) serum PAF levels were directly correlated with anaphylaxis severity, (ii) serum PAF acetylhydrolase activity was inversely correlated with anaphylaxis severity and (iii) PAF acetylhydrolase activity was significantly lower in patients who had fatal anaphylactic reactions to peanuts than in controls (Vadas *et al*, 2008).

In addition to basophils, neutrophils (Jönsson *et al*, 2011, 2012) and monocytes (Strait *et al*, 2002) have also been reported to play critical roles in the onset of anaphylaxis in the murine system. In these pathways, FcγRs, IgG and PAF are involved. However, the exact players, antibody class(es) and the chemical mediators in the human system remain to be determined. Nevertheless, extensive studies are needed before a final conclusion is reached on the IgG-, FcγRs- and PAF-mediated pathways in the human system.

## Allergen-independent pathways

An alternative, putative mechanism underlying allergic transfusion reactions is that biological response modifiers (BRMs), such as inflammatory cytokines and chemokines, accumulate in blood components during storage, are infused along with transfused blood and result in allergic reactions (Fig 1). Stored PLT concentrate supernatants (PC-SNs) accumulate striking levels of BRMs during storage, including vascular endothelial growth factor, soluble CD40 ligand, histamine, transforming growth factor-β1 and RANTES (Wadhwa *et al*, 1996; Edvardsen *et al*, 1998; Phipps *et al*, 2001; Wakamoto *et al*, 2003; Garraud *et al*, 2012). There is reason to believe that these molecules are infused at what may be clinically significant doses and possibly alter a recipient's immune functions. Although the roles of these BRMs in the onset of allergic reactions remain largely unknown, it is possible that these or other substances induce or modulate allergic reactions.

## Patient factors other than allergens and antibodies

It is known that sera from some patients with chronic idiopathic urticaria have detectable histamine-releasing activity (HRA). Based on this fact, Azuma *et al* (2009a) published a unique report on patient factors in allergic reactions. They noted HRA-like activity, which is the ability to induce Ca^2+^ influx into cultured mast cells (CaIA), in the pre-transfusion sera of patients with transfusion reactions. This suggested that CaIA may be attributed to adverse reactions, particularly urticaria-like manifestations (Azuma *et al*, 2009a). Because a calcium influx is known to precede histamine release from mast cells (Ozawa *et al*, 1993; Baba *et al*, 2008), they speculated that a serum sample showing CaIA activity could potentially induce histamine release, although in small amounts, and that mast cells may be pre-activated to a certain degree beforehand and tend to degranulate when an allergenic blood component is transfused.

This group also encountered another transfusion reaction case that suggested the presence of patient factors. This was a life-threatening reaction with hypotension that occurred after transfusion of fresh frozen plasma containing CD36 (Nak) isoantibodies (Morishita *et al*, 2005). These CD36 antibodies exhibited PLT-activating capability mediated though FcγRIIa, and, interestingly, PLTs from healthy human subjects exhibited a considerable degree of heterogeneity in their responsiveness to this plasma. The PLT surface expression levels of CD36 and FcγRIIa and their degree of binding to these antibodies were apparently associated with the profound differences in PLT responsiveness to this plasma (Wakamoto *et al*, 2005).

## Passive transfer of antibodies and passive sensitization

### Passive transfer of antibodies

In a study of IgA-deficiency among 73,569 blood donors, Vyas *et al* (1975) found 113 donors who were IgA-deficient. Of these, 13 had class-specific or high-titre IgA antibodies. However, no noticeable adverse reactions were observed (Vyas *et al*, 1975). In another study, Winters *et al* (2004) reviewed 22 apheresis PC transfusions derived from four IgA-deficient donors with IgA antibodies and observed no allergic reactions. Therefore, passive transfer of IgA antibodies may not elicit a transfusion reaction. To date, no reports on the effects of passive transfer of Hp antibodies have been published. Therefore, the risk of immediate-type allergic reactions elicited by passive transfusion of plasma protein antibodies appears to be very low.

### Passive sensitization

When IgE antibodies against certain allergens, usually foods, inhalant allergens or drugs, are infused into a patient as part of a transfusion, the patient's mast cells and basophils will capture the infused IgE antibodies via the FcεRs expressed on these cells' surfaces. This is so-called passive sensitization. Allergic reactions can occur after a patient ingests or inhales these allergens. One study found that 23% of donors had significant levels of IgE antibodies to common allergens (Johansson *et al*, 2005a). Therefore, the risk of passive sensitization by blood and plasma transfusion is probably not low.

The same group of investigators subsequently performed an *in vivo* study in which two units (each approximately 300 ml) containing known concentrations of IgE antibodies to timothy grass pollen (8–205 kilo units antigen (kUA)/l) were selected and transfused into patients (Johansson *et al*, 2005b). IgE in the transfused plasma could be detected in the circulation of the recipients within 3 h after transfusion; the mean half-life of IgE was 1·13 d. Basophil sensitization was obvious in the 3 h sample, and it increased rapidly to a peak after 3·4 d and then declined over days to weeks. This indicated that IgE antibodies to certain allergens could sensitize patient basophils.

Actual cases of passive sensitization have also been reported. Branch and Gifford (1979) reported generalized urticaria in a recipient who received cephalothin 2 d after a whole blood transfusion from four donors. One of these donors was allergic to cephalothin and had developed antibodies against this antibiotic. Cephalothin antibodies were identified in the recipient's blood after the transfusion, but not before the transfusion.

Recently, a unique case of passive transfer of nut allergy after transfusion of fresh frozen plasma (FFP) was reported (Arnold *et al*, 2007). A non-atopic female patient received a transfusion of two FFP units. Two days later, she ate a muffin and some peanut butter and developed throat tightness, dyspnoea, dysphagia and a pruritic rash within minutes. One week later, a skin prick test for peanut protein was positive, and peanut-specific IgE levels were 2·7 kU/l (normal levels: <0·35 kU/l). Two months later, a second skin prick test was negative and peanut-specific IgE was undetectable. No reaction was observed following a supervised oral peanut challenge test 3 months after her transfusion. One of the FFP units was traced back to a whole blood donor with a history of peanut allergy and anaphylaxis. A subsequent peanut skin prick test for that donor was positive, and her peanut-specific IgE levels were >100 kU/l.

## Laboratory tests

Plasma protein levels and plasma protein antibodies against IgA and Hp of both the IgG and IgE classes, if possible, need to be examined first. In-house systems are typically used to detect these deficiencies and antibodies, although commercial systems recently became available for detecting IgA deficiency and IgA antibodies (Palmer *et al*, 2012). Even without identifying the allergens and antibodies, it is possible to examine whether transfused blood products were causative and whether the reactions were allergic in nature on the basis of the following two tests.

### Tryptase test

Tryptase is the most abundant secretory granule-derived serine proteinase contained in mast cells. Elevated tryptase levels in serum, plasma and other biological fluids are consistent with mast cell activation in systemic anaphylaxis and other immediate hypersensitivity allergic reactions (Schwartz *et al*, 1987, 1995). In the above-mentioned anaphylaxis case that occurred after passive transfer of peanut allergens to a sensitized boy, the serum tryptase levels after this reaction were significantly high and suggested a causative relationship between the reaction and the transfusion (Jacobs *et al*, 2011). In two of three recent anaphylactic reaction cases that occurred after transfusions of methylene blue-treated plasma, serum tryptase levels were elevated and aided in the diagnoses (Dewachter *et al*, 2011; Nubret *et al*, 2011). In Japan, serum tryptase levels are measured in patients suffering from non-haemolytic transfusion reactions if an allergic reaction is suspected and the patients' serum samples are available. It has been shown that the tryptase test is useful in diagnosing allergic transfusion reactions (Hirayama, 2010). However, it has a couple of drawbacks.

First, it is often difficult to obtain a patient's serum sample for a tryptase test because of the short tryptase half-life, which is only 2 h after its release into the circulation (Schwartz, 1990). Second, only a small amount of tryptase is produced by basophils, the other mediator of allergic reactions (Castells *et al*, 1987; Foster *et al*, 2002). In contrast, the basophil activation test (BAT) has no restrictions regarding the timing for patient sample collection and can assess basophil activation.

### Basophil activation test (BAT)

The BAT was recently developed for managing allergic diseases. In this test, a patient's whole blood sample is incubated with an allergen. Subsequent basophil activation is assessed using flow cytometry on the basis of upregulation of cell degranulation/activation markers, CD63 or CD203c (Bühring *et al*, 1999; Boumiza *et al*, 2005). This test was recently applied to transfusion medicine. In the three above-mentioned anaphylactic reaction cases that occurred after methylene blue-treated plasma transfusion, the BAT was positive (Dewachter *et al*, 2011; Nubret *et al*, 2011). All three patients showed a positive reaction to methylene blue and/or patent blue, a methylene blue-related dye that is more antigenic than methylene blue. One patient was also BAT-positive for transfused FFP.

We assessed whether the BAT could be applied to transfusion medicine using PC-SNs from nine cases with allergic transfusion reactions and 12 cases without transfusion reactions (Matsuyama *et al*, 2009). Basophils were obtained from whole blood samples of healthy subjects because the patients' blood was not available. Three of 9 PC-SNs with allergic reactions activated basophils in at least one of five blood samples. The degree of basophil activation was quite different among the blood panels, suggesting the involvement of patient factors in the onset of allergic reactions. In contrast, only one of 12 PC-SNs without allergic reactions elicited basophil activation; this was probably a false BAT-positive result because that PC-SN was BAT-positive only to one of the five blood samples. Furthermore, the extent of CD203c up-regulation was minimal and close to the cut-off point. These data suggest that the BAT may be a useful tool for examining allergic transfusion reactions.

Allergic transfusion reactions are usually diagnosed on the basis of symptoms and their time of onset (immediate or after transfusion). Tests for plasma protein deficiency and antibodies are not always performed. Even if these tests are performed, they are usually negative. Therefore, in many allergic reaction cases, there is no evidence other than that the reactions were truly allergic in nature and the transfusion was causative. If a BAT is performed using residual transfused blood and the patient's blood is positive, it may be possible to conclude that the transfused blood elicited the reaction. Unfortunately, the BAT has only just begun to be used in transfusion medicine. Therefore, its usefulness has not yet been fully evaluated. In addition, its sensitivity and specificity remain to be determined (Hirayama, 2010). Therefore, similar but larger-scale studies are required to make a final evaluation of the BAT.

## Prevention

### Pre-transfusion medications

Acetaminophen, a representative antipyretic, and diphenhydramine, a well-known anti-histaminic, are widely used as pre-transfusion medications to prevent transfusion reactions, despite the lack of evidence regarding their preventative effects.

Kennedy *et al* (2008) conducted a large, prospective, randomized, double-blind controlled trial of acetaminophen and diphenhydramine as pre-transfusion medications for preventing transfusion reactions versus a placebo and focused on the two major reaction types: febrile and allergic. Study medications were administered 30 min before the transfusions. Patients were monitored for reaction symptoms that developed within 4 h of the transfusion. A total of 315 eligible haematology/oncology patients were enrolled. Of these, 62 developed transfusion reactions while receiving a total of 4199 transfusions. Twenty-nine reactions occurred in patients who received the active drug and who had received a total of 2008 transfusions (1·44/100 transfusions), while 33 reactions occurred in patients who received the placebo and who had received a total of 2191 transfusions (1·51/100 transfusions). The majority of these reactions (36/62) were urticarial in nature and occurred at the same rate between the active drug and placebo groups. However, there was a limitation to this study; it was unknown whether diphenhydramine was useful for preventing recurrent reactions because patients with a history of allergic reactions were excluded.

Sanders *et al* (2005) performed a retrospective study of 7900 transfusions administered to 385 paediatric patients with cancer or who required haematopoietic stem cell transplantations. The incidence of allergic reactions was 0·75%. Allergic reactions were associated with 0·9% transfusions in patients who were administered diphenhydramine compared with 0·56% in those patients who were not administered this drug.

These two reports marginally support the common practice of pre-transfusion medication, particularly for chronically transfused patients. One drawback is that diphenhydramine may not be sufficiently strong to prevent allergic reactions. However, this assumption remains unconfirmed because the effects of stronger pre-medications, such as steroids, remain unknown.

### Plasma-reduced (or concentrated) PLTs and washed PLTs

Although it remains unknown whether allergic reactions after PLT transfusions are caused by plasma proteins and their associated antibodies or BRMs, it has been noted that decreased amounts of plasma decreases the risk of allergic reactions. Decreasing the amount of plasma in PLT preparations can be achieved in three ways. One is to use PLTs from several buffy coats, which are pooled and supplemented with a solution other than plasma. This decreases the residual plasma content to 30–40% of the total volume (plasma-reduced PLTs). The second approach is to use apheresis PLTs in which highly concentrated PLTs are prepared using an apheresis device such as Trima Accel (Daskalakis *et al*, 2012; Johnson *et al*, 2012) or Amicus (Vassallo *et al*, 2010) with (plasma-reduced PLTs) or without (concentrated PLTs) adding a supplemental solution. The third approach is to use both pooled and apheresis PLTs. As much plasma as possible is removed using centrifugation or a cell processor, such as the Cobe 2991, after which PLTs are re-suspended in a supplemental solution (washed PLTs).

Using these methods, the residual plasma amounts with washed PLTs are usually <10%. Five studies evaluated plasma-reduced PLTs, concentrated PLTs and washed PLTs for their preventative effects against allergic transfusion reactions. These results are summarized in Table I. The conclusions drawn from these studies are as follows:

1. The preventative effects of plasma-reduced or concentrated PLTs are significant, although they may be limited.
2. Washed PLTs are more effective than plasma-reduced or concentrated PLTs.
3. Residual plasma after PLT washing should be <10% or 20 ml to achieve highly preventative effects.

**Table I:** Summary of studies investigating the effectiveness of plasma-reduced and washed PLT transfusion

							Findings			
							
Study	Study type	Pooled or apheresis	Plasma-reduced or washed	Residual plasma content	Additional Solution	Patients (n)	Cases/allergic reactions per transfusion (n)	Transfusion reactions	Timing of transfusion after preparation	Other observations
de Wildt-Eggen *et al* (2000)	Randomized	Pooled	Plasma-reduced	Approximately 30%	PAS-II	21	Control: 5/192 plasma-reduced: 0/132	Total reactions: 23/192 (12%) in control vs. 7/132 (5·3%) in plasma-reduced group, *P* < 0·05; allergic: 5/192 in control vs. 0/132 in plasma-reduced group, *P* < 0·05.	Up to 4 d. but no information about how long preventive effects last after preparation.	PLT count recovery: no information
										1-h and 20-h CCI after plasma-reduced PLT transfusion were slightly, but significantly lower than CCI after control PLT transfusion.
Tobian *et al* (2011)	Observational	Apheresis	Plasma-reduced (concentrated)	Less than 33%	None	91	Control: 160/3 193 concentrated: 23/3 326	Concentrated PLTs reduced allergic reactions in 91 of 135 patients (67%) who had developed significant or multiple allergic reactions. 160/3 193 (5·0%) in control vs 23/3 326 (0·7%) in concentrated, *P* < 0·001.	No information	PLT count recovery and CCI: see Karafin *et al* (2012) below
										Washed RBCs also significantly reduced allergic reactions to RBCs.
			Washed by Cobe 2 991	No information	Saline	44	Control: 89/1 413 concentrated: 50/1 001 washed: 12/2 857	Concentrated PLTs failed to reduce allergic reactions in 44 of 135 patients (33%) who had developed significant or multiple allergic reactions. 89/1 413 (6·3%) in control vs 50/1 001 (5·0%) in concentrated, *P* = 0·176. Transfusion with washed PLTs significantly reduced allergic reactions [12/2 857 (0·4%), *P* < 0·001].		
						44	Control: 57/969 washed: 9/1 225	Washed PLTs reduced allergic reactions in 44 patients who had developed severe or life-threatening allergic reactions.		
								57/969 (5·9%) in control vs 9/1 225 (0·7%) in washed, *P* < 0·001		
Karafin *et al* (2012)	Observational		Plasma-reduced (concentrated)	Less than 33%	None					PLT count recovery: 79% (*P* < 0·001).
										0-1-h CCI was comparable, but 20-h CCI was reduced by 25% (*P* < 0·03).
			Washed by Cobe 2991	No information	Saline					PLT recovery: 80% (*P* < 0·001).
										1-h and 20-h CCI were reduced by 21% and 41%, respectively (*P* < 0·001)
Silvergleid *et al* (1977)	Observational	Pooled	Washed by centrifugation	Less than 1%	Hand-made solution*	6	Incidence is not clearly described	Massive ulticaria, extensive ulticaria, and severe serum sickness were prevented.	Immediately	PLT count recovery: more than 90 - 95%
Buck *et al* (1986)	Observational	Pooled & apheresis	Washed by Cobe 2991	94% for pooled PLT	Saline	6	Control: no information washed: 0/207	6 patients with a history of severe allergic reactions received 207 washed PLTs, and showed no further allergic reactions.	Within 4 h	PLT count recovery: 88%. 1-h and 24-h CCI were comparable between the two groups. No effects on febrile reaction
Azuma *et al* (2009b)	Observational	Apheresis	Washed by centrifugation	<20 ml (<10%)	M-sol† (hand-made)	12	Control: 117/276 washed: 1/156	12 patients developed 117 reactions (mostly allergic reactions) after unwashed 276 PLT transfusions. Only one minor allergic reaction was reported after 156 washed transfusions.	Within 1 d	PLT count recovery: 87% (*n* = 75) Satisfactory CCI and no clinically evident bleeding were observed in washed groups.
2009b										

Plasma-reduced: 60-70% of plasma was removed, and additional solution was supplemented.

Concentrated: 60-70% of plasma was removed, but additional solution was not supplemented.

Washed: more than 90% of plasma was removed.

Control: unmanipulated PLT.

* Sodium Citrate: 10 mM, citric acid: 5 mM, dextrose: 209 mM, albumin: 0·7 mM.

† 77 mM NaCl, 3 mM KCl, 1 mM CaCl2, 21 mM Na acetate, 15 mM glucose, 9·4 mM Na3 citrate, 4·8 mM citric acid, 44 mM HaHCo_3_, 1.6 mM MgSO_4_.

PLT, platelet; CCI, corrected count increment.

However, there are some limitations to using washed PLTs. First, washed PLTs are not prepared by blood centres as an approved blood component in any country. Second, washing PLTs is time-consuming; this hampers the number of washed PLTs that can be prepared at one time. Blood centres require high throughput automated devices to produce washed PLTs as an approved blood component. Third, washing weakens the antimicrobial/microbicidal activity of plasma (Hirayama *et al*, 2011). Fourth, the duration for which the preventative effects last after washing remains unknown. Although these effects seem to last for a day after preparation (Azuma *et al*, 2009b), the duration of their effectiveness after that remains unclear. Fifth, it is uncertain whether plasma proteins or BRMs, which are probably released from platelet granules, cause allergic reactions. If BRMs are responsible, they would re-accumulate in the PLT components and washing would no longer be effective after a certain point. Sixth, platelet recovery after washing is 80–95% of unwashed PLTs, and the corrected count increment (CCI) may be slightly lower in washed PLTs than in unwashed PLTs, as shown in Table I. Seventh, PLTs may be activated. PLT activation may lead to up-regulation of CD62, resulting in shorter *in vivo* survival, and enhance the release of BRMs from PLTs, which may cause adverse reactions.

These issues need to be resolved before blood centres begin to routinely supply high-quality washed PLTs. Regarding the last limitation, additive solutions have been developed that can attenuate PLT activation. Adding magnesium and potassium ions to the commercial additive solutions PASII and PASIII completely prevented PLT activation, as evidenced by comparable levels of CD62p expression, BMR release, glucose consumption and lactate production (de Wildt-Eggen *et al*, 2002; Gulliksson *et al*, 2003; Shanwell *et al*, 2003). In addition, M-sol, which contains magnesium and potassium ions, reportedly has attenuating effects on PLT activation (Hirayama *et al*, 2007). Although there has been no reported study of washed PLTs re-suspended in PASII and PASIII containing magnesium and potassium ions, a study of washed PLTs re-suspended in M-sol has been reported (Azuma *et al*, 2009b; Table I). Detailed evaluations of each additive solution are needed before making any final conclusions on this issue.

Recently, the Japanese Society of Transfusion Medicine and Cell Therapy issued guidelines (http://www.jstmct.or.jp/jstmct/Document/Guideline/Ref9-2.pdf) for washed PLTs and their preparation in hospitals. These guidelines recommended M-sol as a supplemental solution (Hirayama *et al*, 2007). However, a solution that is made in-house, such as M-sol, is not approved for producing approved blood components. This issue also needs to be resolved before blood centres prepare washed PLTs as an approved blood component.

### Transfusions to IgA- or Hp-deficient patients

Transfusions for IgA- and Hp-deficient patients require special attention. FFP should be IgA- or Hp-free. In Japan, blood centres screen for IgA- and Hp-deficient donors and store a sufficient number of FFP preparations for IgA- and Hp-deficient patients. Deficient donors are registered for FFP donations only. IgA- or Hp-free PLTs and RBCs are usually supplied as washed components to deficient patients. RBCs can be washed up to 3 times to remove as much plasma as possible; however, it is difficult to perform the same procedure with PLTs. In these cases, PLTs may be obtained from registered donors.

### Immune tolerance induction to IgA-deficient patients with IgA anaphylactic reactions

Common variable immunodeficiency (CVID) involves low levels of most or all of the immunoglobulin classes, a lack of B lymphocytes or plasma cells that can produce antibodies and frequent bacterial infections. Patients with CVID frequently develop IgA antibodies that may induce anaphylaxis after intravenous immune globulin (IVIG) preparations or blood transfusions are administered. These patients may develop tolerance to IgA if IgA-depleted IVIG preparations are used. Ahrens *et al* (2007) induced tolerance in five CVID patients with a history of anaphylaxis by infusing IgA-depleted IVIG preparations that contained only small amounts of IgA, until IgA antibody activity was decreased significantly or became undetectable. Subsequently, all five patients developed complete tolerance to IVIG preparations. Although the precise mechanism underlying tolerance is unknown, the most plausible explanation is that the infused residual IgA molecules in IVIG preparations block IgA antibodies and that high-dose IVIG inhibits IgA antibody production. However, more studies are needed before drawing any final conclusions on this treatment.

## Treatment

Although pre-transfusion medications are not effective, most transfusion reactions are easily treated (Geiger & Howard, 2007; Roback *et al*, 2011). When urticaria occurs, diphenhydramine may be administered. Severe urticarial reactions may require treatment with methylpredonisolone or predonine. Once a severe reaction develops or anaphylaxis occurs, prompt action should be taken to maintain oxygenation levels and stabilize hypotension. Epinephrine may be administered intramuscularly or subcutaneously. If the patient is unconscious or in shock, epinephrine may be given intravenously. If bronchospasm is present, respiratory symptoms may not respond to epinephrine, and adding a beta II agonist or aminophylline may be required.

## Conclusions and future perspectives

Plasma protein deficiencies and antibodies are not always evaluated in cases of allergic reactions. Even when evaluated, they are rarely identified; therefore, the cause of the allergic reaction is not specified and the causative relationship between the reaction and the transfusion remains obscure in many cases. This is usually not a problem because allergic reactions are generally self-limiting and respond well to treatment in many cases. However, in life-threatening cases, the pathoaetiology needs to be determined to prevent these reactions during the next transfusion. Underlying diseases, medicines and infections may elicit symptoms like those of allergic reactions. To confirm the causative relationship between a reaction and a transfusion, the tryptase test and the BAT should be useful, although the BAT has only just begun to be used in transfusion medicine, and its usefulness has not been fully evaluated. These tests only assess the involvement of mast cells and basophils in allergy. Because studies on the murine immune system suggest the involvement of neutrophils and monocytes in allergy, tests that assess their involvement may need to be developed.

Regarding recurrent cases, allergic reactions are usually preventable by using washed PLTs and RBCs. Although these practical techniques are beneficial for patients, a fundamental solution in which the exact mechanism of a reaction is identified should be sought, and prevention should then be based on this underlying mechanism.

One of the keys to a solution may be obtained by determining the duration for which washed PLTs retain allergic reaction-preventing capabilities after the washing procedures. If washed PLTs prove to be effective long enough to allow the re-accumulation of BRMs after washing, the BRM-mediated, allergen-independent pathway would be disproved and the allergen-dependent pathway would be affirmed. We would then need to search for plasma protein deficiencies and antibodies other than those we have identified. In addition, an extensive search for other allergens, like food allergens, should be made, although the importance of food allergens is currently quite controversial. In contrast, if washed PLTs are effective for only a short time after washing, this would indicate that BRMs that accumulate in PLT components are responsible. In this case, we would need to focus on identifying the responsible BRMs and determine how these BRMs induce allergic reactions. This would ultimately lead to the development of new strategies for the prevention and treatment of allergic reactions.
